# Mitochondrial Features of Mouse Myoblasts Are Finely Tuned by Low Doses of Ozone: The Evidence In Vitro

**DOI:** 10.3390/ijms24108900

**Published:** 2023-05-17

**Authors:** Chiara Rita Inguscio, Elisa Dalla Pozza, Ilaria Dando, Federico Boschi, Gabriele Tabaracci, Osvaldo Angelini, Pietro Maria Picotti, Manuela Malatesta, Barbara Cisterna

**Affiliations:** 1 Department of Neurosciences, Biomedicine and Movement Sciences, University of Verona, I-37134 Verona, Italy; chiararita.inguscio@univr.it (C.R.I.); elisa.dallapozza@univr.it (E.D.P.); ilaria.dando@univr.it (I.D.); barbara.cisterna@univr.it (B.C.); 2 Department of Engineering for Innovation Medicine, University of Verona, I-37134 Verona, Italy; federico.boschi@univr.it; 3 San Rocco Clinic, I-25018 Montichiari, Italy; tabaracci@sanrocco.net (G.T.); osva.ange@virgilio.it (O.A.); 4 Lab of Move, I-37135 Verona, Italy; pmpicotti@labofmove.it

**Keywords:** medical ozone, reactive oxygen species, mitochondrial cristae, nuclear factor erythroid 2-related factor 2 (Nrf2), fluorescence microscopy, transmission electron microscopy

## Abstract

The mild oxidative stress induced by low doses of gaseous ozone (O_3_) activates the antioxidant cell response through the nuclear factor erythroid 2-related factor 2 (Nrf2), thus inducing beneficial effects without cell damage. Mitochondria are sensitive to mild oxidative stress and represent a susceptible O_3_ target. In this in vitro study, we investigated the mitochondrial response to low O_3_ doses in the immortalized, non-tumoral muscle C2C12 cells; a multimodal approach including fluorescence microscopy, transmission electron microscopy and biochemistry was used. Results demonstrated that mitochondrial features are finely tuned by low O_3_ doses. The O_3_ concentration of 10 μg maintained normal levels of mitochondria-associated Nrf2, promoted the mitochondrial increase of size and cristae extension, reduced cellular reactive oxygen species (ROS) and prevented cell death. Conversely, in 20 μg O_3_-treated cells, where the association of Nrf2 with the mitochondria drastically dropped, mitochondria underwent more significant swelling, and ROS and cell death increased. This study, therefore, adds original evidence for the involvement of Nrf2 in the dose-dependent response to low O_3_ concentrations not only as an Antioxidant Response Elements (ARE) gene activator but also as a regulatory/protective factor of mitochondrial function.

## 1. Introduction

Low doses of gaseous ozone (O_3_) applied as oxygen (O_2_)–O_3_ mixtures are successfully administered as a complementary treatment for several diseases [1,2,3,4]. Consistently with the principle of hormesis, i.e., “the beneficial effect of a low-level exposure to an agent that is harmful at high levels” [5], the positive outcome of low O_3_ doses relies in the cascade of molecular events that are triggered by an initially induced moderate oxidative stress which activates an antioxidant cell response without causing damage [1,6]. Mild O_3_ treatment stimulates the expression of the Antioxidant Response Elements (ARE)-driven genes via the nuclear factor erythroid 2-related factor 2 (Nrf2)-mediated Kelch-like ECH-associated protein1 (Keap1)-dependent pathway [7,8,9]. The Nrf2 protein stability is tightly regulated: under basal conditions, Keap1 targets Nrf2 for continuous ubiquitination and proteasomal degradation in the cytoplasm [10,11,12]. Keap1 is a critical sensor of oxidative stress [13], and its chemical modification [14,15] or the direct disruption of the Keap1-Nrf2 binding interface [16,17] prevents the degradation of Nrf2 that translocates to the nucleus [18], where it promotes the transcription of ARE-driven genes [7,19]. Several lines of evidence showed that, among its cytoprotective activities, Nrf2 mediates the maintenance of cell redox homeostasis by regulating and sustaining the structural and functional integrity of the mitochondria [20]. Accordingly, Nrf2 has been demonstrated to associate with mitochondria [21,22].

Mitochondria, essential cell organelles involved in more than just adenosine triphosphate (ATP) synthesis [23,24], are known to be sensitive to even mild oxidative stress [25], thus representing a susceptible target for O_3_. However, studies focused on the effects of low O_3_ concentrations on mitochondria are still scarce [26,27].

In the present study, we investigated the response of muscle cell mitochondria to the mild oxidative stress induced by 10 or 20 µg O_3_/mL O_2_, as these concentrations are widely used for medical treatments. Skeletal muscle represents a primary target in the clinical application of O_3_, and we used the immortalized non-tumoral cell line, C2C12 as an in vitro model suitable to apply refined techniques under strictly controlled experimental conditions. An integrated multimodal approach including cytochemistry and immunocytochemistry at fluorescence and transmission electron microscopy and biochemical assays provided original information on the O_3_ dose-dependent modifications of mitochondria’s fine morphological and molecular features.

## 2. Results

### 2.1. Cell Viability

**Figure 1:**
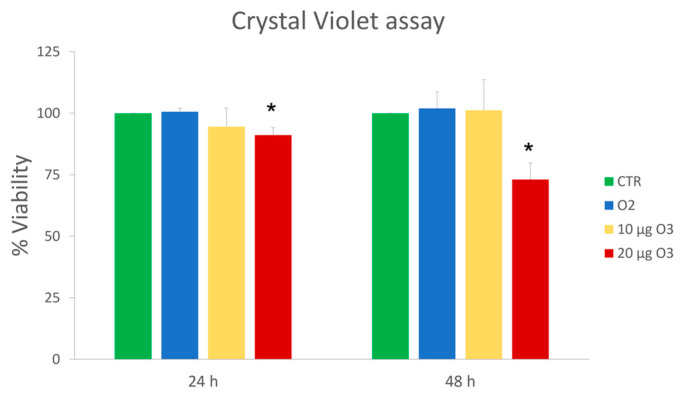
Mean values ± standard error of the mean of cell viability at 24 h and 48 h after treatment (*n* = 15). (*) indicates the statistically significant difference compared to the respective control (CTR).

C2C12 cells were treated with O_2_–O_3_ mixtures at concentrations of 10 µg or 20 µg O_3_; pure O_2_ was also used to discriminate the effects of O_3_. Cell viability was evaluated at 24 h and 48 h after treatment through the Crystal violet assay (Figure 1). Data are representative of 3 independent experiments. 10 µg O_3_ did not affect cell viability at 24 h and 48 h post-treatment, whereas 20 µg O_3_ induced a significant decrease in cell viability at 24 h (*p* = 0.04), which became more substantial at 48 h (*p* = 0.01).

Therefore, 10 µg O_3_ proved safe for murine myoblasts, whereas 20 µg O_3_ decreased cell viability medium and long after gas exposure.

### 2.2. Reactive Oxygen Species (ROS) Production

**Figure 2:**
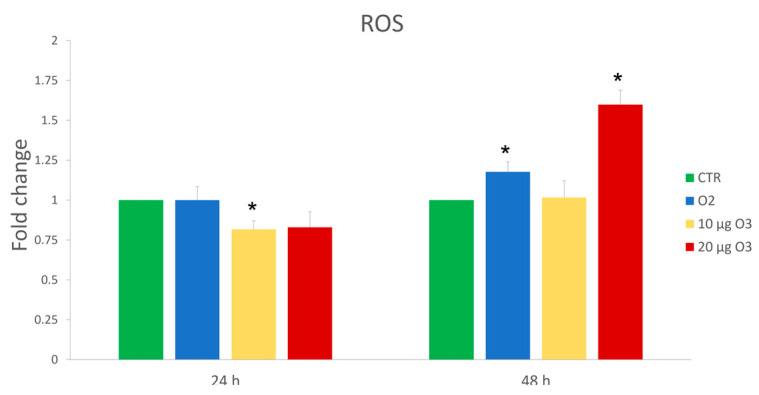
Mean values ± standard error of the mean of Reactive Oxygen Species (ROS) production after 24 h and 48 h of treatment (*n* = 15). (*) indicates the statistically significant difference compared to the respective control (CTR).

The effect on the ROS production was evaluated at 24 h and 48 h after gas treatment through the 2′,7′-dichlorofluorescin diacetate (DCF) probe (Figure 2). Data are representative of 3 independent experiments. At 24 h, a significant decrease in ROS production was found in 10 µg O_3_-treated cells (*p* = 0.02). However, at 48 h, there was no change in the amount of ROS in 10 µg O_3_-treated cells, whereas, in O_2_-treated cells, a significant increase in ROS production was found (*p* = 0.04) that was even greater in 20 µg O_3_-treated cells in comparison with control (*p* = 0.002).

In sum, 10 µg O_3_ not only did not increase ROS production but even reduced their amount, probable thanks to the well-known antioxidant response to eustress [5]. Conversely, 20 µg O_3_ induced a marked increase of ROS a long time after gas exposure, similar to that induced by pure O_2_, maybe due to excessive oxidative stress that the cell could not compensate for.

### 2.3. S-Phase Evaluation

**Figure 3:**
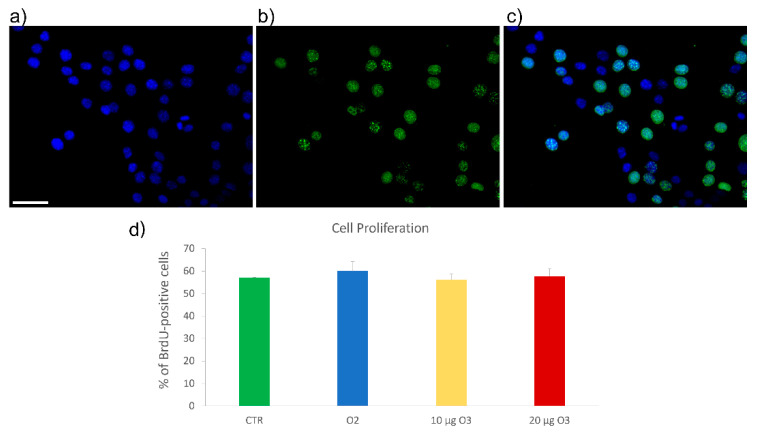
Representative images of C2C12 cells immunolabelled for 5-Bromo-2′-deoxyuridine (BrdU) (**a**), counterstained for DNA with Hoechst 33342 (**b**); merged image in (**c**). Bar: 50 μm. (**d**) Mean values ± standard error of the mean of percentages of BrdU-positive cells 24 h after the treatment (*n* = 30). No significant difference was found among the cell samples. CTR: control.

5-Bromo-2′-deoxyuridine (BrdU) is a thymidine analogue incorporated into newly synthesized DNA during the S-phase, thus labeling proliferating cells and allowing their detection after specific immunostaining. BrdU-positive cell nuclei were green-fluorescing, while nuclear DNA was counterstained in blue with Hoechst 33342 (Figure 3). Data are representative of 3 independent experiments. 24 h after gas exposure, no significant difference (*p* = 0.5) was observed in the percentage of BrdU-positive cell nuclei in the four samples (control, O_2_-, 10 μg O_3_-, 20 μg O_3_-treated cells).

The absence of alteration in cell proliferation rate supports the notion that the decrease in cell viability observed after treatment with 20 μg O_3_ using the Crystal violet assay is due to increased cell death.

### 2.4. Mitochondrial Membrane Potential Assay

**Figure 4:**
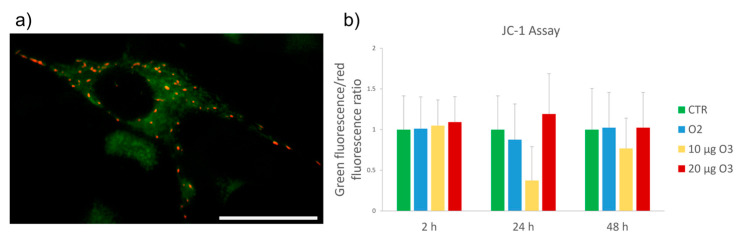
(**a**) Representative image of JC-1 staining. Bar: 50 μm. (**b**) Mean values ± standard error of the mean of green/red fluorescence ratio at 2 h, 24 h and 48 h after treatment (*n* = 30). No significant difference was found among the cell samples at all time points. CTR: control.

Mitochondrial membrane potential, a key parameter to evaluate the mitochondrial function, was monitored using JC-1 dye, which accumulates in mitochondria where it emits either red or green fluorescence depending on the membrane potential: the red signal indicates polarized (highly functional) mitochondria, the green signal the depolarized (less functional) ones (Figure 4a). In addition, the green-to-red fluorescence ratio was evaluated by digitally quantifying the fluorescent signal (Figure 4b). This method not only allowed signal quantification but also gave information about cell morphology, mitochondrial morphology, and intracellular distribution. Data are representative of 3 independent experiments. No significant differences were detected in the cell samples at all time points (2 h, 24 h and 48 h post-treatment, *p* = 0.9772, *p* = 0.127 and *p* = 0.7422, respectively).

This suggests that no significant alteration in mitochondrial respiratory activity was induced by gas treatment under our experimental conditions.

The high variability found in all samples is likely due to the physiologically high intra- and intercellular heterogeneity in mitochondrial activity.

### 2.5. Ultrastructural Analysis

**Figure 5:**
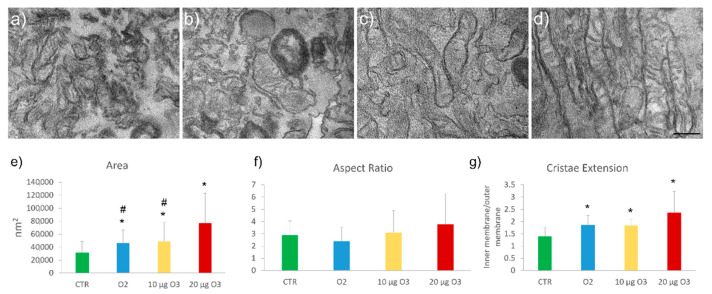
Transmission electron micrographs of control (CTR) (**a**), O_2_- (**b**), 10 μg O_3_- (**c**) and 20 μg O_3_- (**d**) treated cells. Bar: 200 nm. Mean values ± standard error of the mean of mitochondrial area (**e**), aspect ratio (**f**), and cristae extension (**g**) (*n* = 30). (*) indicates the statistically significant difference in comparison with the respective CTR. (#) indicates the statistically significant difference in comparison with 20 µg O_3_.

The fine morphology of mitochondria was investigated through transmission electron microscopy. All samples showed well-preserved elongated mitochondria rich in lamellar cristae (Figure 5a–d).

Morphometric analysis (data are representative of 2 independent experiments) showed a significant increase of mitochondrial area in O_2_- (*p* = 0.014), 10 μg O_3_- (*p* = 0.049) and 20 μg O_3_- (*p* < 0.001) treated cells (Figure 5e) in comparison with the untreated controls. No difference was found between O_2_- and 10 μg O_3_-treated cells (*p* = 0.88), but both samples showed values significantly lower than 20 μg O_3_-treated cells (*p* = 0.01 and *p* = 0.03, respectively). The cristae extension, expressed as the ratio between the inner and outer mitochondrial membrane, increased in O_2_- (*p* = 0.001), 10 μg O_3_- (*p* < 0.001) and 20 μg O_3_- (*p* < 0.001) treated cells compared to the control (Figure 5g). The aspect ratio, assessed as an index of mitochondrial elongation, showed no significant difference among the cell samples (Figure 5f).

In sum, all gas treatments increased mitochondrial size and cristae extension, probably due to the high amount of available O_2_, but 20 μg O_3_ caused a significantly higher mitochondrial swelling compared to 10 μg O_3_. On the other hand, gas treatments did not affect the mitochondrial shape.

### 2.6. Nrf2 Distribution

**Figure 6:**
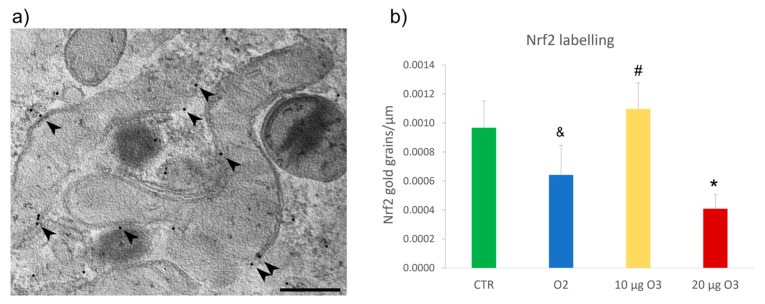
(**a**) Representative transmission electron micrograph of C2C12 cell after immunolabelling of nuclear factor erythroid 2-related factor 2 (Nrf2) (arrowheads) (**a**). Bar: 200 nm. (**b**) Mean value ± standard error of the mean of anti-Nrf2 labelling 24 h after treatment (*n* = 30). (*) indicates the statistically significant difference compared to the control (CTR) sample. (#) indicates the statistically significant difference in comparison with 20 µg O_3_; (&) indicates the statistically significant difference with 10 µg O_3_.

The mitochondrial localization of Nrf2 was evaluated by ultrastructural immunocytochemistry, which revealed Nrf2 binding to the outer membrane (Figure 6). Data are representative of 2 independent experiments. The Nrf2 immunolabelling density, expressed as the number of gold grains per membrane length unit (μm), was significantly lower in 20 μg O_3_-treated cells compared to the control (*p* = 0.02). Moreover, 10 μg O_3_-treated cells showed substantially higher immunolabelling density than O_2_-treated (*p* = 0.007) and 20 μg O_3_-treated (*p* = 0.002) samples.

Therefore, 20 μg O_3_ caused a marked reduction of mitochondria-associated Nrf2 compared with 10 μg O_3_, confirming the involvement of Nrf2 in mitochondrial protection under oxidative stress conditions [20].

## 3. Discussion

In the present study, the effects of exposure to low O_3_ doses on the structural and functional features of C2C12 cells were investigated with a focus on mitochondria as target organelles for the mild oxidative stress induced by O_3_.

Ten μg O_3_ was safe for all time points, consistent with our previous data on other cell types [9,18,28,29,30]. Conversely, 20 μg O_3_ induced a significant decrease in cell viability at both 24 h and 48 h after treatment. Accordingly, after treatment with 20 μg O_3_ a decreased cell viability characterized by a high level of lactic dehydrogenase (as necrosis marker) and increased caspase activity was described in peripheral blood mononuclear cells [26]. Twenty μg O_3_ was found to increase the death rate also in human adipose-derived adult stem cells [30].

Consistently with similar findings obtained in other cell lines [4,18,28], low doses of O_3_ did not affect the percentage of C2C12 cells in the S-phase, indicating that the decreased viability observed in these cells was not due to O_3_-mediated alteration in cell proliferation.

It is known that impaired mitochondrial function and decreased ATP production play a crucial role in cell death [26]. In C2C12 cells, the fine morphology of mitochondria was affected by both O_2_ and O_3_ treatments. At 24 h after the exposure, a mitochondrial area significantly increased in O_2_- and 10 μg O_3_-treated cells compared to untreated cells, suggesting that the mitochondrial enlargement was due to the exposure to O_2_ rather than to O_3_. Similarly, mitochondria swelling was observed in hyperoxia-injured alveolar cells [31,32]. In 20 μg O_3_-treated cells, an even more significant enlargement of mitochondria was observed (significantly higher than in 10 μg O_3_-treated cells), suggesting that in these samples, the increase in size was also due to O_3_. It is known that mitochondrial swelling may also occur following oxidative stress [33,34].

Interestingly, the mitochondrial enlargement was not associated with shape (aspect ratio) alterations in all gas-treated samples. Therefore, although it has been reported that oxidative stress can induce mitochondrial fragmentation [35], under our mild experimental conditions, no mitochondrial modifications were found, which might suggest fusion or fission events.

Independently of the mitochondrial size, cristae extension significantly increased in C2C12 cells treated with O_2_ or both O_3_ doses. Such an increase in the inner membrane length likely represents a response to the increased O_2_ availability. Furthermore, the cristae density is considered a predictor of maximum oxygen uptake in relation to mitochondrial volume [36]. However, the increase in length of mitochondrial cristae was not paralleled by marked modifications of the mitochondrial membrane potential, i.e., the electrochemical gradient generated by the electron transport chain driving ATP synthesis, considered an index of mitochondrial function.

O_3_, when reacting with unsaturated fatty acids, leads to the production of ROS, mainly hydrogen peroxide, accompanied by the formation of lipid ozonation products (LOPs) [37]. The production of ROS should be counterbalanced by levels of antioxidant enzyme activity sufficient to maintain cellular redox homeostasis. Interestingly, at 24 h from the gas exposure, 10 μg O_3_-treated cells showed a significant decrease in ROS amount, highlighting the rapid antioxidant response promoted by this O_3_ concentration. This finding is consistent with the increased expression of Heme oxygenase 1 (*Hmox-1*) observed in other cell types treated with the same O_3_ concentration [4,8,9]. Conversely, a significant increase of ROS was found in 20 μg O_3_-treated and O_2_-treated cells 48 h after gas exposure. This finding suggests that the concentration of 20 μg O_3_ and pure O_2_ promote oxidative stress at longer times that the cell cannot fully compensate, as discussed below.

Nrf2 is a transcription factor regulating the expression of a network of antioxidant enzymes [7]. Its activity is finely controlled by Keap1, which is a direct target of ROS [21,38]. Modifying Keap1 cysteine residues by oxidative signals prevent the continuous proteasomal degradation of Nrf2, promoting immediate Nrf2 translocation to the nucleus where the Nrf2-mediated transcription of ARE-driven antioxidant genes takes place [39]. Interestingly, Nrf2 and Keap1 localize in the mitochondrial outer membrane forming a ternary complex with the Keap1-binding protein phosphoglycerate mutase family member 5 [21,22]. This finding, obtained by immunofluorescence [21] and mitochondria sub-fractionation [22], was confirmed in the present study by ultrastructural immunolabelling, which specifically detected the Nrf2 protein on the mitochondrial outer membrane of all cell samples. On this membrane, Nrf2 has been proposed to directly impact mitochondria preservation [22], serving as a sensor of mitochondrial function and ROS production [38]. Mitochondrial function is, in turn, closely related to the level of ROS [25]. Under stress conditions, the Nrf2-Keap1 pathway is thus crucially involved in the functional modulation of mitochondria [20,40,41], regulating both mitochondrial and cytosolic ROS production [40].

Interestingly, the density of Nrf2 immunolabelling on the mitochondrial outer membrane was significantly reduced in 20 μg O_3_-treated cells in comparison with the control, and 10 μg O_3_-treated cells and a similar reduction of Nrf2 immunolabelling was also observed in O_2_-treated cells in contrast with 10 μg O_3_-treated cells. Previous evidence showed that a lack of Nrf2 results in an increase of ROS [20] as well as an increased sensitivity to mitochondrial membrane permeability transition and mitochondrial swelling [22]. Therefore, the decrease of mitochondria-associated Nrf2 in 20 μg O_3_-treated cells could be related to the marked organelle swelling. However, this structural alteration does not significantly affect the organelle function, as demonstrated by the absence of changes in membrane potential. Therefore, it is likely that the mitochondrial alterations observed in 20 μg O_3_-treated cells represent one of the oxidative stress-related impairments that collectively lead to cell death.

## 4. Materials and Methods

### 4.1. Cell Culture and Treatment

C3H mouse muscle myoblasts C2C12 (ECACC 91031101) were grown in Dulbecco’s modified Eagle’s medium (DMEM), supplemented with 10% heat-inactivated fetal bovine serum (FBS), 2 mM L- glutamine, 1% penicillin/streptomycin, 0.5% amphotericin-B (all reagents were purchased from Gibco, Waltham, MA, USA) at 37 °C in a 5% CO_2_ humidified atmosphere in T75 flasks.

The cells were exposed to O_2_–O_3_ gas mixtures produced by an OZO2 FUTURA apparatus (Alnitec, Cremosano, CR, Italy) from medical-grade O_2_; the apparatus allows photometric real-time control of O_3_ concentration and gas flow rate. O_3_ was used at the concentrations currently administered in the clinical practice for intramuscular injections, i.e., 10 and 20 µg O_3_/mL O_2_. In addition, these concentrations proved to be non-toxic for various cultured cells and tissues [4,8,9,18,28,29]. Pure O_2_ was administered to cells to discern the effect elicited by O_3_ from O_2_, as gas treatment is dispensed as a mixture of the two. Control cells underwent the same handling without exposure to the O_2_–O_3_ gas mixture or pure O_2_.

When sub-confluent (80%), the cells were detached using 0.25% trypsin/EDTA (Gibco, Waltham, MA, USA) and seeded for specific analyses. For Crystal violet cell viability assays and ROS evaluation, samples of 4 × 10^6^ cells were suspended in a 10 mL medium in a 20 mL polypropylene (O_3_ resistant) syringe (Terumo Medical Corporation, Somerset, NJ, USA). An equal volume of gas (10 mL) was then collected into the syringe (so that the final gas pressure corresponded to the atmospheric one) through a sterile filter (Alnitec s.r.l.) to avoid contamination. The sample was gently and continuously mixed with the gas mixture for 10 min since it has been ascertained that during this period, cell samples react with the ozone dose totally [42]. The cells were then seeded in a 96-multi-well plate after gas treatment and analyzed. For the S-phase assessment, morphological and morphometrical analyses at transmission electron microscopy, and the evaluation of mitochondrial membrane potential, cells were seeded on glass coverslips (24 × 24 mm), allowed to adhere for 24 h and then subjected to gas exposure by placing the coverslips into a 50 mL polypropylene syringe containing 20 mL culture medium. The syringe was then connected to the OZO2 Futura output valve, and an equal volume (20 mL) of gas was collected through a sterile filter (Alnitec s.r.l.). After gently moving for 10 min to dissolve the gas in the medium, the coverslips were taken out of the syringe, placed in wells containing fresh culture medium and incubated for the appropriate times for the analyses.

### 4.2. Cell Viability Assay

C2C12 cells were plated in 96-well plates (1 × 10^4^ cells/well) after the treatment. After 24 or 48 h the cells were washed with phosphate buffer saline (PBS) and stained with a crystal violet solution (Sigma-Aldrich, St. Louis, MO, USA). The dye was solubilized in PBS containing 1% sodium dodecyl sulfate (SDS) and measured photometrically (A595 nm) to determine cell viability.

### 4.3. Measurement of Reactive Oxygen Species (ROS) Production

The non-fluorescent 2′,7′-dichlorofluorescin diacetate (DCF) probe, which becomes highly fluorescent in reaction with ROS, was used to evaluate cellular ROS production. Briefly, C2C12 cells were plated in 96-well plates (1 × 10^4^ cells/well) after treatment. After 24 or 48 h the cells were incubated in a culture medium without FBS with 10 μM DCF (Sigma-Aldrich) at 37 °C for 20 min. The medium with DCF was removed, and the cells were incubated with culture medium at 37 °C for 10 min. The cells were then washed with Dulbecco’s PBS buffer (ThermoFisher, Waltham, MA, USA), and fluorescence was measured by using a multimode plate reader at 485/535 nm (TECAN Infinite^®^ M Nano PLUS, Männedorf, Switzerland). The values were normalized on cell viability.

### 4.4. S-Phase Evaluation

The S-phase evaluation assessed the cell proliferation rate in C2C12 cells 24 h after gas exposure. Cells (6 × 10^4^ cells per 24 × 24 mm slides) were pulse-labelled with 20 µM BrdU (Sigma-Aldrich) at 37 °C for 30 min and then fixed with 70% ethanol. To partially denature DNA, cells were incubated with 2 N HCl for 20 min at room temperature, then neutralized for 3 min with 0.1 M sodium tetraborate (pH 8.2) (Sigma-Aldrich), washed with PBS and permeabilized for 15 min with PBS containing 0.1% bovine serum albumin and 0.05% Tween-20 (Sigma-Aldrich). Cells were then incubated with a mouse monoclonal antibody direct against BrdU (BD Diagnostics, Franklin Lakes, NJ, USA) diluted 1:20 in PBS for 1 h, washed in PBS and incubated with Alexa Fluor 488-conjugated anti-mouse secondary antibody (Molecular Probes, Invitrogen, Milan, MI, Italy) diluted 1:200 for 1 h, washed with PBS twice and stained for DNA with 0.1 µg/mL Hoechst 33342 (Abcam, Cambridge, UK) in PBS for 10 min. Samples were finally mounted with PBS/glycerol 1:1 solution. BrdU-positive cell percentage was assessed in 30 randomly selected fields (40×) for each experimental condition. Observation of cell samples was performed using an Olympus BX51 microscope (Olympus Italia S.r.l., Segrate, MI, Italy) equipped with a 100 W mercury lamp under the following conditions: 450–480 nm excitation filter (excf), 500 nm dichroic mirror (dm), and 515 nm barrier filter (bf) for Alexa Fluor 488; 330–385 nm excf, 400 nm dm, and 420 nm bf, for Hoechst 33342. Images were acquired with QICAM Fast 1394 Digital Camera (QImaging, Surrey, BC, Canada) and processed with Image-Pro Plus software (Media Cybernetics, Inc., Rockville, MD, USA).

### 4.5. Mitochondrial Membrane Potential Assay

The mitochondrial membrane potential (ΔΨ_m_) of C2C12 cells was assessed by using the lipophilic cation JC-1 ((ThermoFisher). Cells (7 × 10^4^ cells per slide) were stained after 2 h, 24 h and 48 h from gas exposure with JC-1 (final concentration 2 µM) for 15 min in a sterile incubator at 37 °C with 5% CO_2_ in the dark, and then briefly washed twice with PBS. Cell samples were observed using an Olympus BX51 microscope (Olympus Italia S.r.l., Segrate, MI, Italy) equipped with a 100 W mercury lamp, with the following conditions: excitation filter (400–500 nm), emission (≥520 nm). Images were recorded with an Olympus Camedia C-5050 digital camera (Olympus Italia S.r.l.). JC-1 fluorescence was assessed in 20 randomly selected fields (20×) for each experimental condition. In addition, a routine was written in MATLAB (2018b version, Mathworks) to quantify red and green fluorescence-positive pixels and the ratio between green and red fluorescence signals were expressed.

### 4.6. Transmission Electron Microscopy

Ultrastructural morphometric and immunocytochemical analyses were carried out at transmission electron microscopy to investigate the effects of exposure to low O_3_ concentrations on mitochondria features and Nrf2 mitochondrial location. The cells (2 × 10^4^ per 22 mm slides) were treated with the gas. After 24 h, the cells were fixed for 1 h at 4 °C with 2.5% glutaraldehyde and 2% paraformaldehyde in 0.1 M phosphate buffer pH 7.4, washed and post-fixed at 4 °C for 30 min with 1% OsO_4_. Cells were then dehydrated with acetone and embedded in Epon resin as a monolayer [43] (all reagents were purchased from Electron Microscopy Sciences, Hatfield, PA, USA). For ultrastructural morphology and morphometry, ultrathin sections were collected and stained with Reynolds lead citrate (Electron Microscopy Sciences) for 2 min. For immunocytochemistry, ultrathin sections were collected and immunolabelled. Briefly, sections were floated on normal goat serum diluted 1:100 in PBS for 3 min, incubated overnight at 4 °C with the anti-Nrf2 antibody (Abcam #ab62352, Cambridge, UK) diluted 1:2 with PBS containing 0.1% bovine serum albumin (Fluka, Buchs, Switzerland) and 0.05% Tween 20. Sections were then floated on normal goat serum as above and then incubated for 30 min with a goat anti-rabbit IgG secondary antibody conjugated with 12-nm gold particles (Jackson ImmunoResearch Laboratories Inc., West Grove, PA, USA), diluted 1:20 in PBS. After rinsing with PBS and water, the sections were finally air-dried and weakly stained with Reynolds lead citrate for 1 min. As immunostaining controls, some grids were incubated without the primary antibody and then processed as described above. Observation of the samples was performed through a Philips Morgagni transmission electron microscope (FEI Company Italia Srl, Milan, Italy) operating at 80 kV; image acquisition was made with a Megaview III camera (FEI Company Italia Srl, Milan, Italy). Morphometric analysis of the mitochondrial area, aspect ratio parameter (calculated as the ratio of major mitochondrial axis and minor axis lengths) and the ratio between inner and outer mitochondrial membrane (estimating the extension of cristae independently of the mitochondrial size) was carried out on 20 randomly chosen mitochondria per experimental condition. Quantitation of anti-Nrf2 immunolabelling was performed by estimating the gold grain density on 30 randomly chosen mitochondria (36,000×) per each experimental condition. The gold grains were counted, and the labeling density was expressed as several gold grains/mitochondrial perimeter length (μm).

Based on our previous investigations [7], mitochondria morphometry and Nrf2 density were evaluated at 24 h post-treatment to detect morphological changes and Nrf2 location in mitochondria.

### 4.7. Statistical Analysis

Data for each variable were pooled according to the experimental condition and presented as mean ± standard error of the mean. Statistical significance was assessed by the one-way analysis of variance (ANOVA) test followed, in case of significance, by the *t* test for pairwise comparison with Bonferroni correction. The significance was set at *p* ≤ 0.05.

## 5. Conclusions

Altogether, the results of the present study demonstrate that mitochondrial features are finely tuned by low O_3_ doses. In fact, under our experimental conditions, the O_3_ concentration of 10 μg maintained normal levels of mitochondria-associated Nrf2 (indicating a stabilization of the Nrf2-Keap1 complex on the outer mitochondrial membrane), also promoting the increase of size and cristae extension (which is suggestive of increased mitochondrial respiration). Moreover, 10 μg O_3_ could sustain the balance between the oxidative stress induced by the gas treatment and the antioxidant response, even reducing cellular ROS and preventing cell death. Conversely, in 20 μg O_3_-treated cells, where the association of Nrf2 with the mitochondria drastically dropped, mitochondria underwent more significant swelling than after 10 μg O_3_-treatment, while ROS and cell death increased.

This study, therefore, adds original evidence for the involvement of Nrf2 in the dose-dependent response of cells to low O_3_ concentrations, supporting the notion of its role in ROS control not only as a transcription factor in ARE gene activation but also as a regulatory/protective factor of mitochondrial function. Consistently, previous reports described an O_3_-mediated reduction of mitochondrial damage in rat hearts following ischemia-reperfusion [44] and in rat cochlea and brain following noise-induced hearing loss [45].

The results of the present study have been obtained in an in vitro model, which was especially appropriate for elucidating basic biological mechanisms but cannot fully mimic the several complicating factors occurring in vitro. Moreover, O_3_ effects were investigated in myoblasts, i.e., cultured muscle cells that did not reach the terminal differentiation as the myofibers do in skeletal muscles. Despite such limitations, our findings unequivocally demonstrate that even minor changes in O_3_ concentration differentially affect mitochondria and provide a solid experimental background for further investigation of the molecular mechanisms activated by low O_3_ doses in these organelles. Future research will especially focus on the function of respiratory chain complexes and critical antioxidant enzymes, such as superoxide dismutase and glutathione peroxidase. The knowledge of the relationship between the concentration of the administered O_3_ and the mitochondrial function will be crucial to improve the clinical efficacy of medical O_3,_ especially for intramuscular applications.

## Data Availability

The data presented in this study are available on request from the corresponding author.
